# Investigation into the Feasibility of a Synergistic Photocatalytic Degradation Process for Fracturing Flowback Fluid Streams Utilizing O_3_ and Ti/Ni Composite Materials

**DOI:** 10.3390/molecules30071568

**Published:** 2025-03-31

**Authors:** Huohai Yang, Yeqi Gong, Xin Chen, Renze Li, Yuhang Chen, Mingjun Li, Xinrui Tang

**Affiliations:** 1 School of Petroleum Engineering, Southwest Petroleum University, Chengdu 610500, China; yanghh@swpu.edu.cn (H.Y.); gongyeqiswpu@163.com (Y.G.); 13689612343@163.com (M.L.); 2 Petro China Southwest Oil & Gas Field Company, Chengdu 610051, China; 15082147220@163.com (Y.C.); tangxinruiswpu@163.com (X.T.)

**Keywords:** photocatalysis technology, fracturing flowback fluid, process optimization, wastewater treatment technologies, photocatalytic materials, pollutants

## Abstract

The ecological impact linked to hydraulic fracturing, namely with the usage of water and the energy-intensive disposal of flowback fluids, has led to a thorough evaluation of alternative treatment approaches that are more environmentally friendly. The objective of this work was to create coralline-like anatase TiO_2_/α-Ni(OH)_2_ particles using a hydrothermal approach. The purpose was to improve the efficiency of photocatalysis by increasing the number of oxygen vacancies. An ozone-assisted photocatalytic reaction was used to increase the composite photocatalyst’s degrading efficiency for fracturing flowback fluid. The fracturing flowback fluid’s chemical oxygen demand (COD) degradation efficiency was greatly increased following the introduction of the synergistic treatment system consisting of sedimentation, membrane separation, and ozone photocatalysis. This improvement led to a reduction of 98.42% during a processing time of 90 min, using a Ti/Ni mass ratio of 1:1. This collaborative method partially replaced traditional methods of evaporation concentration and electrochemical degradation, resulting in a 24.18% enhancement compared to individual material catalyst systems. These findings provide crucial insights for improving and optimizing external treatment techniques in shale gas fracturing operations.

## 1. Introduction

Hydraulic fracturing is the main technique used to modify reservoirs in the development of oil and gas. The underlying concept of this technique involves the injection of fracturing fluid into the reservoir, which then exerts pressure on the reservoir to create an intricate network of fractures [1]. Nevertheless, the utilization of hydraulic fracturing will result in a substantial volume of flowback fluid, which contains a significant quantity of hydrocarbons, polymers, additives, and microorganisms. The flowback fluid from fracturing consists mainly of a combination of injected fracturing fluid and fluid from the underground formation. It contains a variety of chemical constituents, including chemical additives from the flowback fluid and organic pollutants, as well as radioactive substances from the underground formation [2]. The release of these compounds onto the Earth’s surface can result in ecological pollution hazards and have an impact on human health [3,4]. Efficiently managing the disposal of fracturing flowback fluid is a crucial issue that must be addressed in order to minimize environmental impact and enhance the economic advantages of oil and gas extraction.

The management of fracturing flowback fluid mainly involves three methods: (a) preparing the liquid for reuse, (b) injecting it into deep wells, and (c) discharging it to fulfil the specified standards (NB/T 14002.3-2015) [5]. “Chemical Oxygen Demand (COD) serves as a critical indicator for assessing organic pollutant levels in wastewater. Elevated COD values in fracturing flowback fluids signify high concentrations of oxidizable organic compounds, posing significant environmental risks if untreated [6]. Recent studies have highlighted the correlation between COD reduction and pollutant removal efficiency, particularly in advanced oxidation processes. For instance, synergistic approaches combining ozonation and photocatalysis demonstrate enhanced COD degradation by promoting hydroxyl radical generation, thereby addressing recalcitrant organic pollutants [7]. When it comes to disposing of flowback fluid from shale gas development in the Sichuan Basin, oil and gas production processes commonly employ a combination of flocculation, membrane filtration, and chemical reaction methods. This approach is used in practical applications to ensure that the disposal of the fluid meets the required discharge standards [8].

In recent years, there have been several past investigations that have specifically examined the actual intricate flowback fluid. For instance, Lin [9] documented a method that involved combining physical and chemical destabilization, filtration, and composite catalytic oxidation to treat fracturing flowback fluid. This resulted in the treated fluid meeting the first-level discharge standard of the Integrated Wastewater Discharge Standard (GB 8978-1996) [10], ensuring its water quality. Tan et al. [11] employed a flocculation–adsorption–membrane filtration–Fenton oxidation technique to treat fracturing flowback fluid. This method effectively circumvents the drawbacks commonly associated with current treatment methods, such as low degradation rates, high economic costs, and secondary pollution. Hence, it is imperative to devise a highly effective oxidation technique for eliminating organic substances from the fracturing flowback fluid, minimizing contamination of membrane and distillation equipment, and assessing its practicality and efficacy in treating actual fracturing flowback fluid [12,13].

Photocatalytic oxidation technology is a cutting-edge and inventive method that effectively prevents the occurrence of secondary pollution, a major problem associated with standard treatment methods. This approach employs semiconductors as catalysts, which are exposed to UV light irradiation. The studied catalytic materials will undergo electron transitions on their surface, resulting in the generation of photogenerated electrons and photogenerated holes [14,15]. Photogenerated electrons possess a significant capacity for reduction, while photogenerated holes possess a significant capacity for oxidation. Both of them have the ability to interact with different functional groups on semiconductor materials, resulting in the effective elimination of organic contaminants from organic wastewater [16,17]. The photocatalytic degradation of fracturing flowback fluid has a rather low level of activity when a single material is employed. Consequently, numerous researchers have endeavored to enhance the efficiency of photocatalytic degradation during the past few decades. Ma et al. [15]. improved the degradation process by modifying photocatalysts, specifically by modifying the BiO/BiOCl structure. As a result, Rhodamine B was degraded by 96.8%. Similarly, Yang et al. enhanced the degradation efficiency by modifying α-Ni(OH)_2_ [18], especially when combined with ozone, resulting in a particularly high degradation efficiency. Liu et al. engineered a CdS-TiO_2_ Z-type heterojunction modified with cobalt-based complexes, optimizing electron transfer pathways to significantly enhance visible-light-driven photocatalytic hydrogen evolution, demonstrating the role of transition metal coordination in charge separation [19]. Umair Baig et al. developed conductive polymer-coated TiO_2_ nanoparticles, revealing enhanced light absorption and dye interaction through spectroscopic and morphological analyses, offering novel strategies for photocatalytic degradation and energy conversion [20]. In recent years, there has been significant research focused on developing new photocatalysts that can harness visible light [21,22,23]. Among these, Ni and TiO_2_ have emerged as highly effective photocatalytic materials and have found widespread application in various photocatalytic degradation processes and have contributed to the understanding and utilization of these materials [24,25,26,27,28,29]. The combination of these two materials holds significant potential for practical applications.

This study employed the utilization of superoxide radicals and hydroxyl radicals, high efficiency, and minimal secondary pollution of photocatalytic technology to address the problems of secondary pollution and high economic costs, which are the main drawbacks of conventional operations [27,29]. In this study, ozone was selected for synergistic catalysis due to its low cost, easy availability, strong oxidizing power, and bactericidal properties, enabling the treatment of fracturing flowback fluids that are difficult to biologically oxidize because of high bacterial content. Consequently, ozone-based treatment of fracturing flowback fluids has been widely adopted in oilfields [30]. Yang’s study findings were utilized to integrate the photocatalytic technology with conventional methods in order to produce a high-quality catalyst, TiO_2_/α-Ni (OH)_2_, on a wide scale. The catalyst is evaluated using the fracturing flowback fluid collected from the Sulige gas field as the degrading object. This study has the potential to address the deficiency in prior research by investigating the actual effectiveness and practicality in real-world applications. Furthermore, we conducted an analysis on the efficacy of photocatalytic materials, as well as the viability and ideal reaction parameters for using the photocatalytic approach in real-world applications. These data could assist us in investigating the process pathway of the photocatalytic approach in degrading fracturing flowback fluid. The aim of this study is to prepare a novel composite photocatalytic material, combine it with ozone oxidation technology, and perform synergistic photocatalytic degradation on on-site fracturing flowback fluid. The on-site treatment process is modularized and simplified into “flocculation sedimentation–filtration–ozone synergistic photocatalytic oxidation with the composite material”, ultimately aiming to reduce treatment time, lower costs, and ensure treatment efficiency.

## 2. Results and Discussion

### 2.1. Creating Technological Process

**Figure 1:**
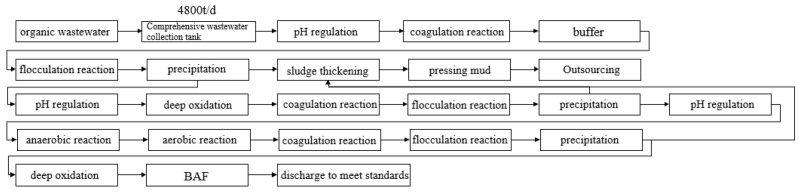
Common processing flow chart on site.

Research indicates that the primary methods currently used for treating fracturing flowback fluid are physical, chemical, and biological procedures [31,32,33,34,35]. Due to the intricate nature of fracturing fluid composition, it is difficult to rely on a single treatment procedure to ensure compliance with emission or compounding criteria. Therefore, a variety of treatment technologies are frequently used to efficiently deal with the different properties of fracturing fluid and guarantee its compliance with regulatory standards. For instance, the usual sequence of steps involved in treating oilfield wastewater at a location in Sichuan, China, might be described as follows: pretreatment, flocculation and sedimentation, filtration of impurities, ion removal, oxidative degradation, and discharge or compounding (Figure 1).

The analysis of the procedure for treating fracturing fluid returns in the statistical example revealed that it involves the utilization of flocculating, settling-membrane filtration, and ozone synergistic photocatalytic oxidation.

#### 2.1.1. Optimization of Flocculating Settling Factors

The first step in the process is the flocculation settling, which serves as the basis for the further advanced treatment of fracturing flowback fluid at oil and gas production sites. During the fracturing process, the fracturing fluid will fully interact with the formation, and as it flows back, it will contain a significant quantity of suspended particulates. The presence of residual suspended solids not only hampers the effectiveness of chemical agents in treatment but also impairs the absorbance of liquids and the efficiency of photocatalytic destruction. Hence, to account for the subsequent deterioration impact and expenses, it is imperative to initially eliminate the suspended solids. Since the main purpose of the flocculating settling steps is to remove suspended particles in wastewater, the performance indicators chosen to evaluate the treatment results of fracturing flowback fluid in this stage are SS and COD.

**Figure 2:**
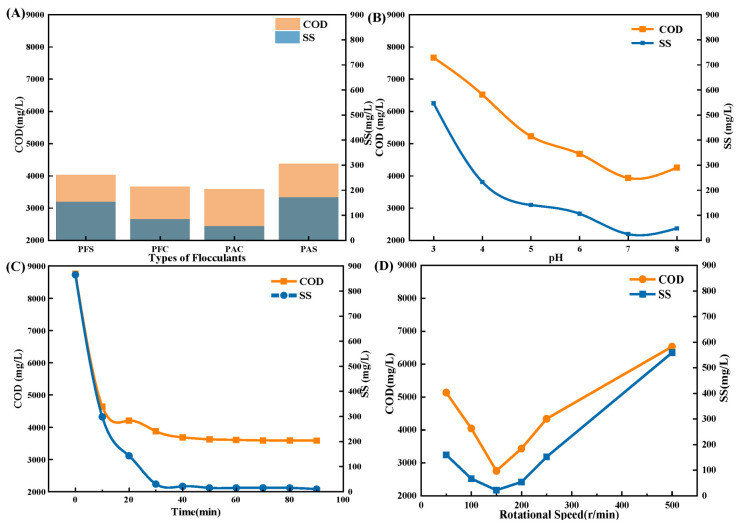
Single factor optimization result figure for flocculating settling. (**A**) Optimal result of the flocculant; (**B**) Optimal selection result of the pH value; (**C**) Optimal result of the time; (**D**) Optimal result of the Rotational Speed.

Based on experimental results (Figure 2), the most effective flocculation treatment occurred at a pH of 7, with a dosage of 2 g/L for PAC, 0.1 g/L for PAM, an agitation intensity of 150 r/min, and a flocculation period of 40 min. The treated fracturing flowback fluid exhibits clarity and transparency, with a COD/COD_0_ ratio of 70% and an SS/SS_0_ ratio of 98.33%.

#### 2.1.2. Study on the Filtration Effect of Microporous Filter Membrane

The filter membrane utilized is a microporous water system composite fiber membrane having a pore diameter of 0.1 μm. Utilize a glass suction filtration apparatus for the purpose of filtration during the filtration process. Resulting in an overall removal COD/COD_0_ ratio of 89.75%.

#### 2.1.3. Optimization of Ozone Aeration Factors

This study employed an ozone-assisted photocatalytic reaction to examine the degradation effect of TiO_2_/α-Ni(OH)_2_ composite photocatalysts on on-site fracturing flowback fluid. The investigation aimed to enhance the degradation effect by utilizing the synergistic effect of ozone aeration.

**Figure 3:**
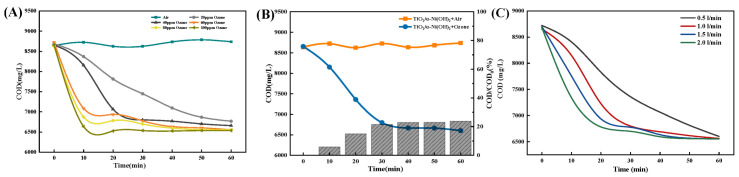
Single-factor optimization result figure for ozone aeration. (**A**) Optimization result of the ozone concentration; (**B**) Optimization result of the ozone aeration time; (**C**) Optimization result of the ozone current velocity.

Based on experimental findings (Figure 3), when treating 100 mL of on-site fracturing flowback fluid with an ozone aeration period of 40 min, a concentration of 40 ppm, and a flow rate of 1 L/min, it is possible to accomplish both degrading efficiency and cost-effectiveness simultaneously. Following the treatment, the ratio of COD/COD_0_ was 24.79%. Nevertheless, this treatment technique is ineffective in removing inorganic salt ions and SS present in the fracturing flowback fluid, and it does not have a noticeable impact on the pH level.

Based on the results of single-factor experiments, appropriate factors and levels were selected, and response surface experiments were designed. The experimental factors and levels are presented in Table 1.

Using COD/COD_0_ as the response value, the experimental results were analyzed by the software Design-Expert 8.0.6.1. The quadratic polynomial model for COD/COD_0_ in terms of time (A), concentration (B), and current Velocity (C) is COD/COD₀ = 24.95 + 2.48·A − 0.53·B + 0.71·C + 0.93·A·B − 0.12·A·C + 0.84·B·C − 6.46·A^2^ − 1.33·B^2^ − 2.62·C^2^ with R^2^ = 0.9946 and R^2^ adj = 0.9876. Response surface plots were generated based on the results.

**Figure 4:**
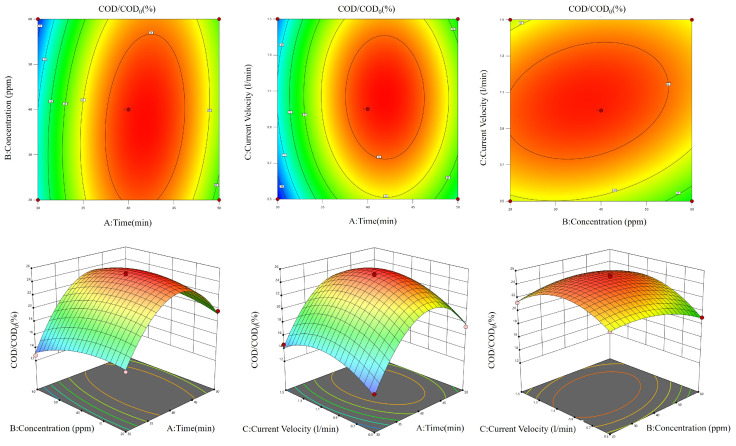
Single-factor optimization result figure for ozone aeration.

Based on the figure (Figure 4), the AB and BC contour maps have an elliptical shape, suggesting a notable interaction between the two parameters (A and B) and (B and C). This indicates that the interplay between the two elements has a significant influence on the COD/COD_0_ values. Additionally, the accompanying 3D surface map exhibits noticeable alterations and sharp peak formations. Simultaneously, the AC response surface exhibits a rather uniform and circular contour map, suggesting that the interplay between the two factors does not have a substantial impact on the COD/COD_0_ values.

Response surface optimization gave the ideal COD/COD₀ values: Duration of 41.84 min, concentration of 38.06 ppm, and current flow rate of 1.06 L/min. To boost the experimental efficiency, we adjusted the conditions to: Duration of 42 min, a concentration of 38 ppm, and a current flow rate of 1.0 L/min. Validation experiments were conducted under these predicted conditions. The validation test yielded a COD/COD₀ ratio of 25.37%, with no significant deviation from the projected value of 25.24%.

#### 2.1.4. Optimization of Photocatalytic Factors

Nano-TiO_2_, α-Ni(OH)_2_, and TiO_2_/α-Ni(OH)_2_ composites were created for experiments to determine the preferred photocatalyst. The factors that were found to have an influence on the results were the duration of exposure to light, the pH level, and the amount of catalyst used.

**Figure 5:**
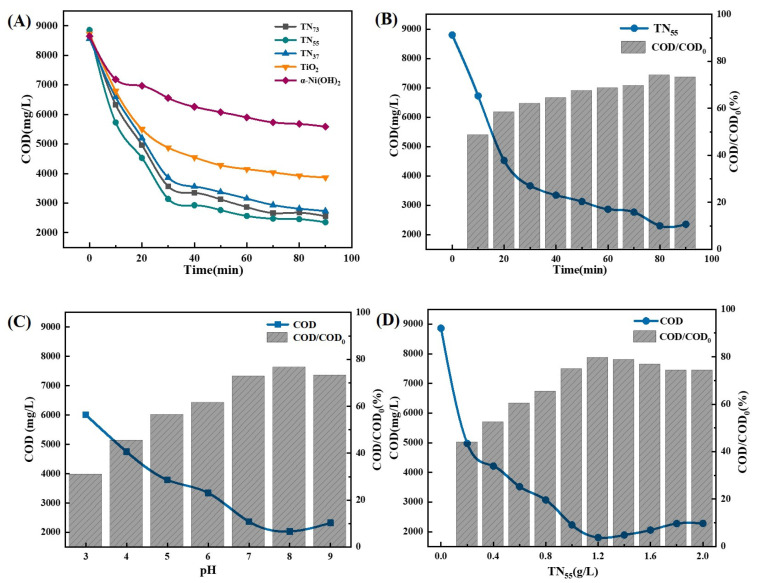
Single-factor optimization result figure for photocatalytic. (**A**) Optimization result of the catalyst; (**B**) Optimization result of the illumination time; (**C**) Optimization result of the pH value; (**D**) Optimization result of the TN_55_ dosage.

Based on the results of the studies (Figure 5), the degrading effect of the materials followed the order TN_55_ > TN_37_ > TN_73_ > TiO_2_ > α-Ni(OH)_2_. Therefore, the TN_55_ composite material was identified as the most effective for treatment when used at a dose of 1.2 g. The duration of the catalytic reaction was 80 min, and the acidity level, as measured by the pH value, was 8. Through the combined action of ozone aeration, the TN_55_ effectively decreased the COD, resulting in a COD/COD_0_ ratio of 80.66%.

Based on the results of single-factor experiments, appropriate factors and levels were selected, and response surface experiments were designed. The experimental factors and levels are presented in Table 2.

**Figure 6:**
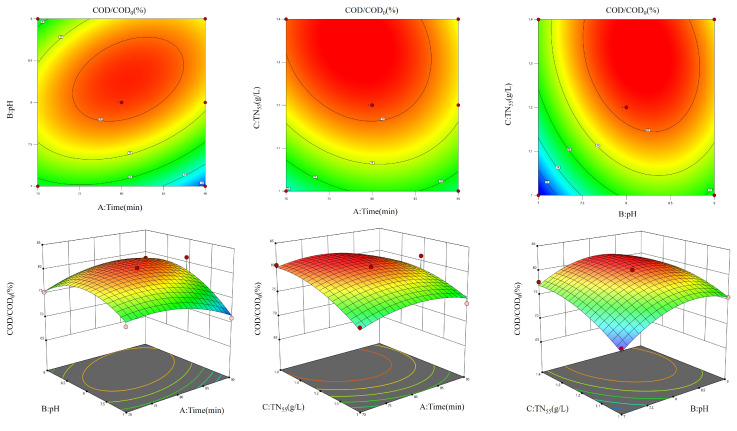
Figure of the combined effect of each factor in Ti/Ni composite material photocatalysis on COD/COD_0_.

Using COD/COD_0_ as the response value, the experimental results were analyzed by the software Design-Expert 13. As illustrated in the figure (Figure 6), the contour plots of AB, BC, and AC exhibited an elliptical shape, indicating that the two interactions were pronounced. This suggests that the interaction of the two factors exerted a considerable influence on the COD/COD_0_ value. Furthermore, the color change and peak steepness of the corresponding 3D surface plots were also more pronounced.

The optimal COD/COD_0_ value was determined using response surface optimization. The duration was 77.95 min, the pH level was 7.74, and the concentration of TN_55_ was 1.16 g/L. To facilitate the experiment, the mentioned conditions were optimized as follows: The duration of the activity was 78 min. The pH level was measured at 8. The concentration of TN_55_ was found to be 1.2 g/L. Validation experiments were conducted under these predicted conditions. The validation test resulted in a COD/COD_0_ ratio of 78.95%, which was not substantially distinct from the projected value of 76.89%.

#### 2.1.5. The Impact of Ozone Aeration on the Efficiency of Ti/Ni Composites in Breaking Down Fracturing Flowback Fluids

**Figure 7:**
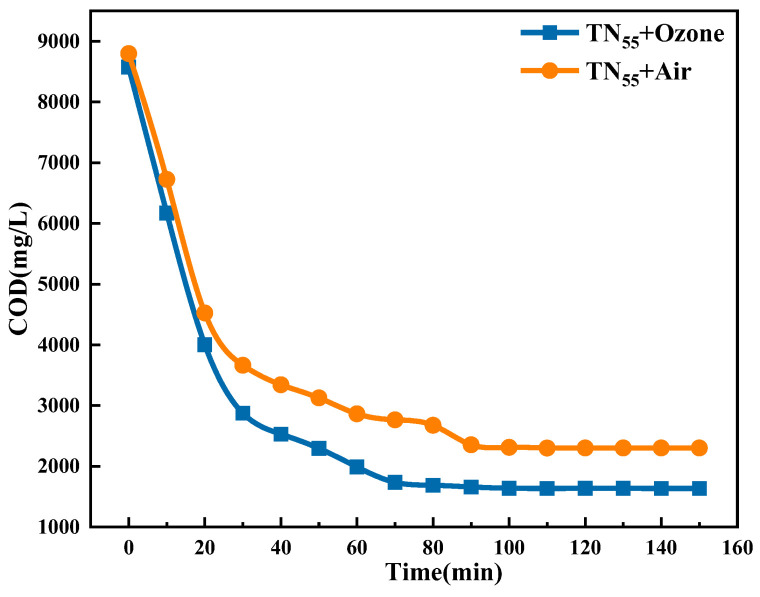
Impact of ozone aeration on degradation efficiency.

The figure (Figure 7) demonstrates that the co-catalyzed ozone and TN_55_ photocatalyst system resulted in a decreased final COD of the fracturing flowback fluid compared to the COD value of the ozone-free co-catalyzed system. When the degradation time was increased to 150 min, it was found that the degradation limit was substantially reached at 90 min, with no significant degradation changes observed in the subsequent 60 min. The COD/COD_0_ ratio is 80.66% under the 90 min co-catalytic condition. This resulted in a 6.42% increase in the degradation rate compared to the condition without ozone. In addition, the ozone co-catalytic process expedited the achievement of the COD degradation threshold, hence improving the degradation efficiency and decreasing the overall degradation duration.

**Figure 8:**
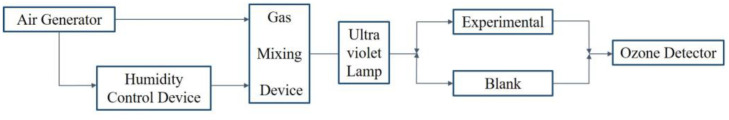
Schematic Diagram of Catalytic Ozone Decomposition Performance Testing Device.

In the preceding section of this study, the necessity of ozone for the entire degradation system was confirmed. To further ascertain the safety and environmental benignity of ozone, an ozone decomposition performance test was executed. This test was designed to verify that ozone would neither induce secondary environmental pollution nor present leakage risks. The testing apparatus is depicted in the figure (Figure 8). The testing conditions were specified as follows: The total gas flow rate was set at 0.3 L/min, the ozone concentration was maintained at 100 ppm, an ozone detector was employed to monitor the real-time ozone concentration, and designated sampling points were selected to construct the resulting diagram, as illustrated below.

**Figure 9:**
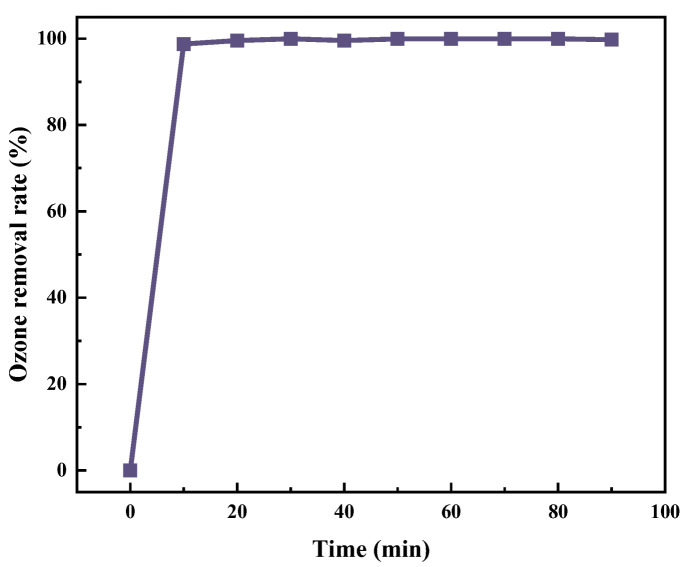
Performance of catalytic ozone decomposition.

According to the analysis of the experimental result figure (Figure 9), the ozone treatment system demonstrates remarkable advantages in both environmental benefits and safety-economic efficiency. From the perspective of the dynamic adsorption process, the ozone removal rate curve exhibits a steep upward trend, indicating that the active adsorption sites are capable of rapidly capturing ozone molecules.

From a degradation perspective, ozone, as a strong oxidizing agent, can effectively decompose recalcitrant organic compounds, addressing the limitations of traditional biological treatment. The synergistic ozone-photocatalytic system achieves a high total COD removal rate (integrating flocculation, membrane filtration, and photocatalysis), significantly outperforming single photocatalysis or conventional methods. Economically, the ozone-synergized technology replaces traditional Fenton and electrochemical oxidation processes, reducing chemical reagent usage and treatment costs while maintaining ease of industrial control over ozone aeration conditions. Environmentally, ozone decomposes into oxygen, generating no secondary pollution.

Throughout the entire design process, despite the fact that the concentration of inorganic salt ions remained persistently elevated, the ratio of COD/COD₀ managed to reach 98.45%. In light of this circumstance, the ozone synergistic photocatalytic treatment technology was implemented to tackle this problem. This particular catalytic treatment technology is especially well-suited for the degradation of COD and functions as a relatively efficient advanced oxidation technique for eliminating stubborn organic contaminants. However, its effectiveness hinges on the simultaneous employment of other methods to remove salt ions. Should this technology be applied on-site for the treatment of fracturing flowback fluid, such an integrated treatment plan must be adopted. Therefore, the ozone-photocatalytic treatment technology is appropriate for decomposing COD, rendering it an advanced oxidation technique for getting rid of difficult-to-degrade organic contaminants (Table 3).

#### 2.1.6. Substitutability of Ozone Synergistic Ti/Ni Composite Photocatalysis for Electrochemical Oxidation

To determine if ozone synergistic photocatalytic technology can replace electrochemical oxidation technology, we selected the electrochemical oxidation conditions that showed the best degradation performance. We then compared the ozone synergistic photocatalytic degradation of TN_55_ composites with the degradation using the same optimized reaction parameters.

**Figure 10:**
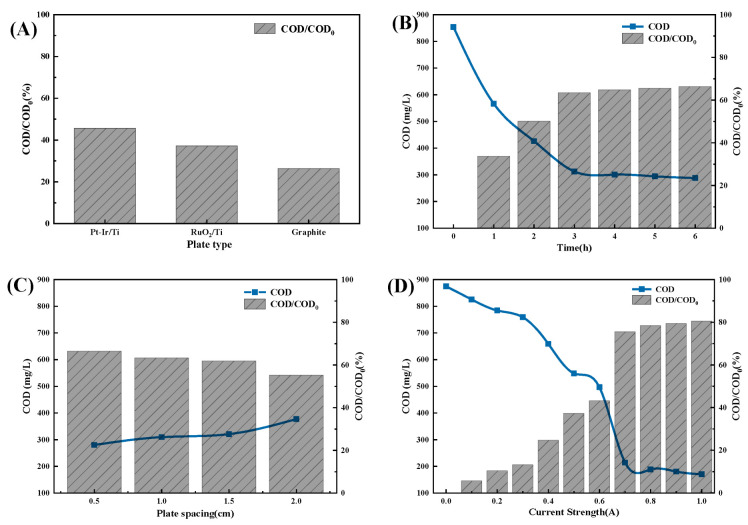
Single-factor optimization result figure for electrochemical oxidation. (**A**) Optimization result of the plate type; (**B**) Optimization result of the electrolysis time; (**C**) Optimization result of the plate spacing; (**D**) Optimization result of the current strength.

Based on the findings from the experiment (Figure 10), the electrochemical oxidation procedure can effectively achieve the intended treatment effect while also decreasing the time and cost of treatment. This was accomplished by choosing the suitable electrode type (Pt-Ir/Ti electrode), maintaining a distance of 0.5 cm between electrodes, adjusting the current intensity to 0.7 A, and setting the electrolysis duration to 2 h. The COD/COD_0_ ratio is 80.46%.

#### 2.1.7. Substitutability Study of Ozone Synergistic Ti/Ni Composite Photocatalysis for Fenton Oxidation

To determine if ozone synergistic photocatalytic technology can replace Fenton oxidation technology, we picked the Fenton oxidation settings that showed the highest degradation performance. The TN_55_ composite ozone synergistic photocatalytic degradation effect was subsequently compared to the ozone synergistic photocatalytic degradation effect while maintaining the same optimized reaction parameters.

**Figure 11:**
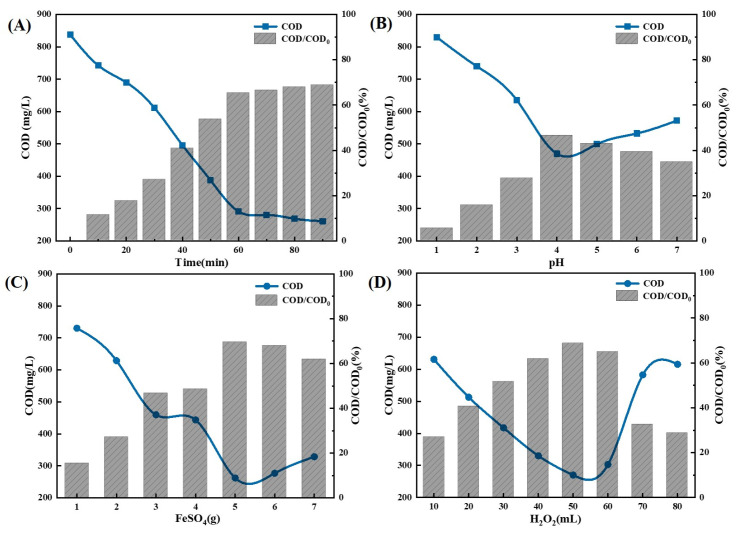
Single-factor optimization result figure for Fenton oxidation. (**A**) Optimization result of the time; (**B**) Optimization result of the pH value; (**C**) Optimization result of the FeSO_4_ dosage; (**D**) Optimization result of the H_2_O_2_ dosage.

Based on the results of the experiments (Figure 11), the Fenton oxidation process, carried out at a pH of 4, with a reaction time of 60 min and a combination of FeSO_4_ and H_2_O_2_ at a ratio of FeSO_4_ 5 g/L + H_2_O_2_ 50 mL/L, achieved an ideal balance between the effectiveness of degradation and cost. The COD/COD_0_ ratio is 70.81%.

As illustrated in the study and the above graph, the ozone synergistic photocatalytic technology achieved a COD/COD_0_ = 83.66%, while the electrocatalytic oxidation technology reached 80.46%. This latter result is slightly inferior to that of ozone synergistic photocatalytic oxidation technology. Finally, the Fenton oxidation technology resulted in a COD/COD_0_ = 70.81%. It can be concluded that ozone synergistic photocatalytic technology is a suitable replacement for Fenton oxidation and electrocatalytic oxidation technology.

### 2.2. Mechanistic Analysis

**Figure 12:**
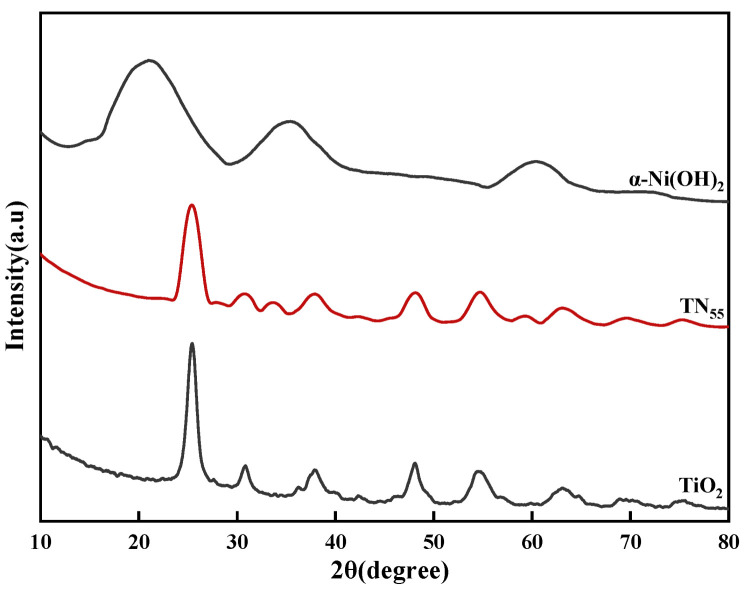
XRD plot for different samples.

X-ray diffraction (XRD) was used to examine the altered microstructure of the materials for the purpose of studying their microstructure. Based on the analysis of the figure (Figure 12), the primary crystal structure of the manufactured TiO_2_ is anatase. This finding, along with the results reported by Chen et al. [36], suggests that the study successfully produced highly pure anatase TiO_2_ photocatalysts. The positions of the diffraction peaks, as determined by the Jade program, align with the standard card PDF #38-0715. This indicates that the α-Ni(OH)_2_ with high purity was successfully synthesized in this experiment. The composites showed minimal changes in the peak positions compared to the two materials, indicating that the synthesis procedure had a limited effect on the crystal structures of TiO_2_ and α-Ni(OH)_2_. This indicates that the α-Ni(OH)_2_ with high purity was successfully synthesized in this experiment. The composites showed minimal changes in the peak positions compared to the two materials, indicating that the synthesis procedure had a limited effect on the crystal structures of TiO_2_ and α-Ni(OH)_2_.

**Figure 13:**
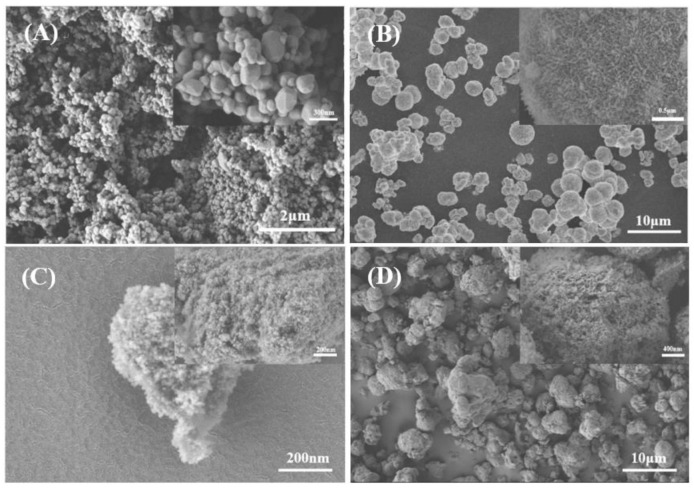
SEM results for (**A**) TiO_2_, (**B**) α-Ni(OH)_2_, (**C**) TN_55_, and (**D**) TN_37_.

Scanning electron microscopy (SEM) was used to study the structural morphology of TiO_2_, α-Ni(OH)_2_, and their composites (Figure 13). (The raw SEM images are in the Supplementary Materials). The microstructure of TiO_2_ consists of multiple tiny TiO_2_ particles that are aggregated into a homogeneous spherical substance. Upon nanoscale examination, it is apparent that the particles exhibit significant variation in size. The particle size is expected to be dispersed between 10 and 150 nm, with an average size of around 50 nm. The α-Ni(OH)_2_ microstructure demonstrates excellent general dispersion, with the exception of some localized aggregation. These aggregates consist of a structured and densely packed collection of short, rod-shaped fluff columns. The microstructure of α-Ni(OH)_2_ exhibits excellent dispersion, with some localized aggregation, and is composed of a uniform and closely packed short rod-shaped columnar structure. The mean diameter of an individual spherical particle is roughly 1 μm. 

The dispersion of TN_55_ is excellent, and it is clear that the material consists of irregular lumps at the micrometer level, with a significant number of TiO_2_ particles at the nanometer level adhering to its surface, displaying a particle size of around 5–10 nm. The TN_37_ has the most efficient dispersion, and its microscopic form is easily distinguishable. The microscopic morphology consists of spherical particles at the micron level, with a particle size distribution ranging from 2 μm to 15 μm. The average particle size is roughly 5 μm. The distribution is reasonably uniform, with a fraction of bigger-sized spherical particles that are greater than 10 μm in diameter. The TiO_2_ particles are included within the network structure created by the α-Ni(OH)_2_ surface protrusions, which evenly coat the initial α-Ni(OH)_2_ protrusions.

**Figure 14:**
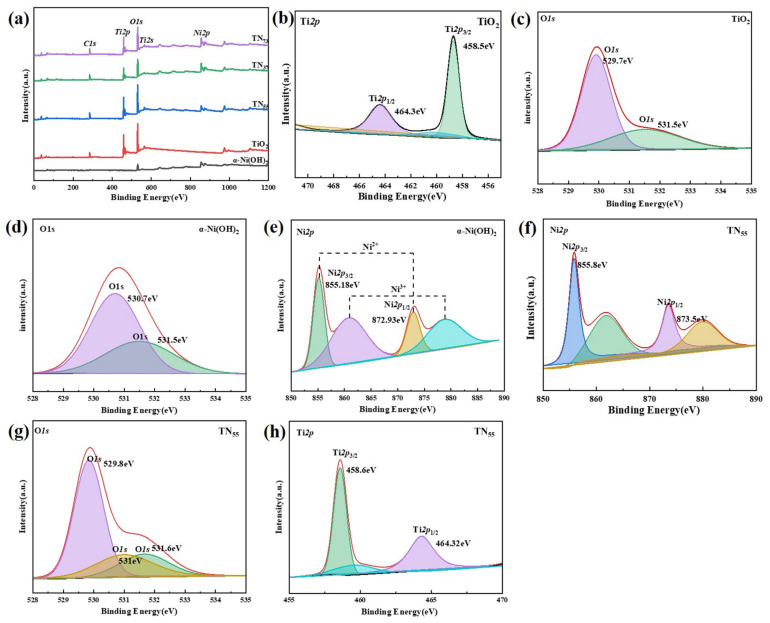
(**a**) XPS plot for different samples. ((**b**,**c**) TiO_2_; (**d**,**e**) α-Ni(OH)_2_ (**f**–**h**) TN_55_).

The Ti2p binding energy diagram of the pure anatase TiO_2_ photocatalyst (Figure 14) shows two peaks. The first peak corresponds to Ti2p_3/2_ with a binding energy of 458.5 eV, while the second peak corresponds to Ti2p_1/2_ with a binding energy of 464.3 eV. The material contains two different kinds of oxygen: lattice oxygen, with a binding energy of 529.7 eV due to the Ti-O-Ti bond, and surface hydroxyl oxygen, with a binding energy of 531.5 eV due to the Ti-OH bond. These exist as a Ti-O-Ti connection. Zhang [37] The binding energies of the Ni2p_1/2_ orbitals in α-Ni(OH)_2_ photocatalysts were measured to be 872.93 eV and 879.0 eV, whereas the binding energies of the Ni2p_3/2_ orbitals were found to be 855.18 eV and 860.8 eV, respectively. The data suggest that Ni ions were present in the forms of Ni^2+^ and Ni^3+^. Furthermore, the materials exhibited binding energies of 530.7 eV and 531.5 eV for the O1s. The first one refers to the O-H link in the hydroxyl group, while the second one refers to the Ni-O bond.

**Figure 15:**
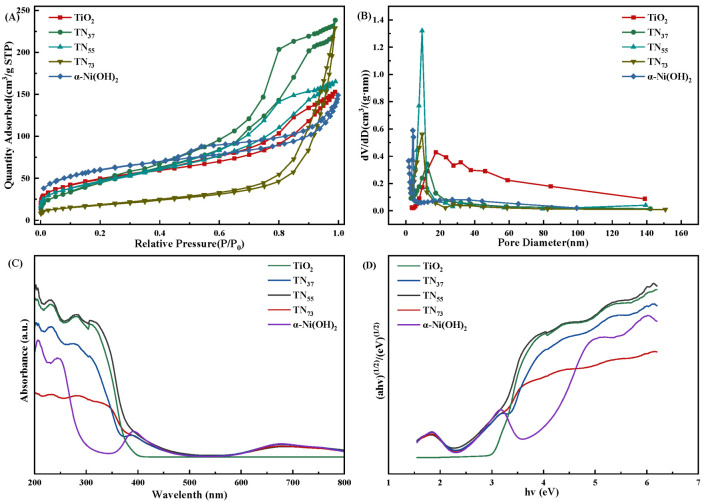
(**A**) photocatalyst adsorption and desorption isotherms. (**B**) BJH pore diameter distribution of different samples. (**C**) UV–vis diffuse reflectance spectroscopy results for different samples; and (**D**) band gaps of different samples.

The composites exhibit photoresponsiveness in the UV-visible range, with minimal variation in the extent of visible light absorption (wavelengths between 400 and 760 nm) (Figure 15A). A spectral region ranging from 600 nm to 800 nm exhibits an absorption band, while a less pronounced absorption shoulder is present in the wavelength range of 360 nm to 450 nm, possibly attributed to the d-d transition of Nickel. The absorption peaks at wavelengths of 232 nm, 283 nm, and 307 nm for all three materials align with those observed in the original TiO_2_, and their locations remain mostly unaltered. The absorption peaks of the three materials exhibited significant variations in height, with TN_55_ > TN_37_ > TN_73_ in terms of strength. This observation suggests that TN_55_ material responds more favorably to light excitation and exhibits superior photocatalytic activity when exposed to light of the same intensity and wavelength. The TN_37_ compound has a faint absorption peak at 388 nm, which is somewhat lower in wavelength compared to the original α-Ni (OH)_2_ absorption peak at 392 nm. This difference in absorption wavelengths results in a blueshift phenomenon [38,39,40]. The forbidden bandwidths of TN_73_, TN_55_, and TN_37_ are 2.5 eV, 2.94 eV, and 2.98 eV, respectively. The forbidden bandwidths of the composites are lower than those of pure TiO_2_ and α-Ni(OH)_2_, indicating that the composites have better oxidation and reduction performances compared to the raw materials. Based on the absorption spectra of ultraviolet light and visible light, the composite material has the ability to absorb a broader range of wavelengths and exhibit superior absorption. Consequently, the photocatalytic performance of the composite material is expected to surpass that of pure anatase TiO_2_ and α-Ni(OH)_2_.

We investigated the N_2_ adsorption-desorption isotherms and pore size distributions of the five samples (Figure 15C). The pore sizes of the four samples were mainly distributed near 10 nm, and the specific surface areas of TiO_2_, α-Ni(OH)_2_, TN_55_, TN_37,_ and TN_73_ were 64.22, 210.66, 176.23, 173.49, and 165.19 m^2^/g, respectively. The average pore sizes are 17.48, 4.33, 9.51, 3.82, and 7.77 nm. The specific surface area and average pore size of these various photocatalysts differ. Among them, α-Ni(OH)_2_ has the highest specific surface area, measuring 210.66 m^2^/g, while TiO_2_ has the lowest specific surface area, measuring 64.22 m^2^/g. Regarding the average pore size, TiO_2_ has the largest average pore size, measuring 17.48 nm, while TN_37_ has the smallest average pore size, measuring 3.82 nm.

However, TN_55_ exhibits the most optimal catalytic effect. It is generally believed that the larger the surface area, the better the adsorption capacity and catalytic effect. However, this does not apply in this case. Possibly, an excessive number of micropores can increase the specific surface area, but it can also hinder the entry of reactants into the pores. For TN_55_, the aperture size and other factors may make it easier for large HPG molecules to enter and react, resulting in the best catalytic effect.

**Figure 16:**
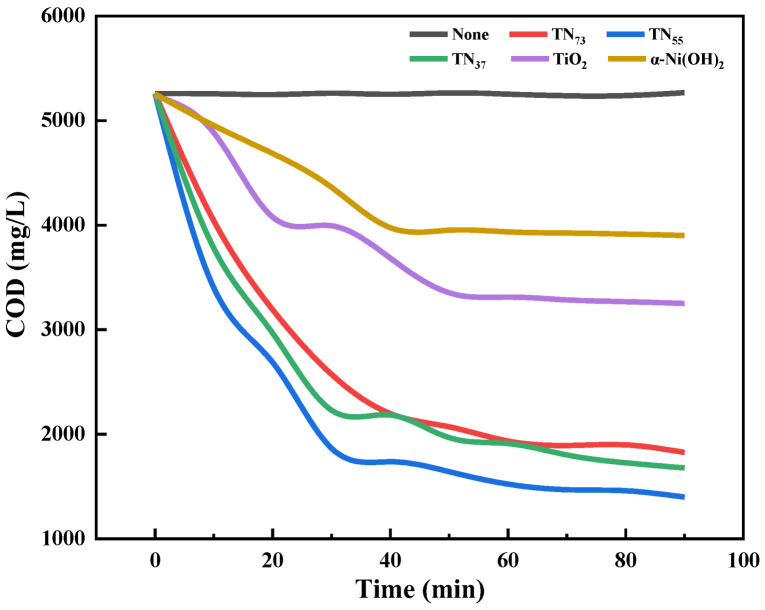
Comparison of the performances of five photocatalysts for the degradation of fracturing flowback fluid.

As illustrated in the figure (Figure 16), the degradation of composite photocatalysts led to a decrease in the COD in comparison to the degradation of nano-TiO_2_ and α-Ni(OH)_2_ solutions. TN_55_ exhibited the highest level of effectiveness, with a degradation rate of 73.4%. The COD of the material in question was lower compared to other materials and had a faster rate of degradation. The TN_37_ yielded a result representing 65.27% of the total, while the TN_73_ group produced a value accounting for 68.11%. The deterioration of the fracturing flowback fluid breakage liquid was observed to occur in the following order: TN_55_ > TN_73_ > TN_37_. TN_55_ showed the most significant degradation, while TN_37_ exhibited the least degradation. Observations were made indicating that the TN_55_ photocatalyst also had the ability to decrease the COD value of the fracturing flowback fluid breakage liquid, suggesting that it demonstrated improved photocatalytic activity under the same experimental conditions. The results of the UV-visible diffuse reflectance spectroscopy studies confirmed this observation.

**Figure 17:**
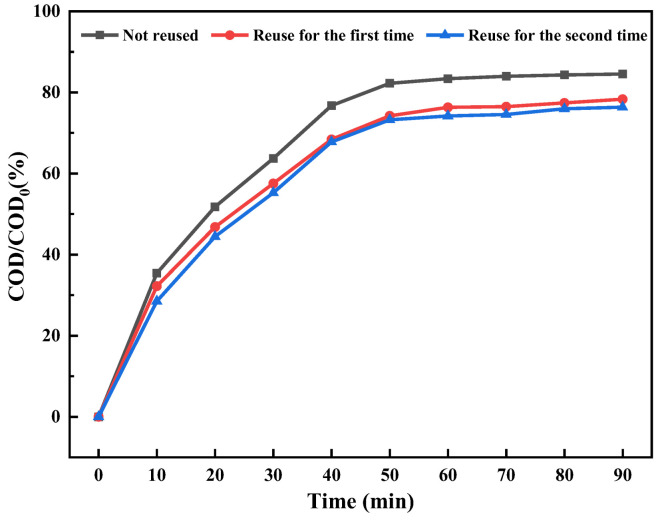
The reusability of TN_55_.

Based on the data presented in Figure 17, the photocatalytic degradation efficiency of fracturing flowback fluid by photocatalyst TN_55_ can reach 84.54% when it is not reused. Upon initial reuse, the photocatalyst TN_55_ demonstrates a remarkable photocatalytic degradation efficiency of 78.33% for fracturing flowback fluid. Upon its second use, the photocatalyst TN_55_ exhibits a photocatalytic degradation efficiency of 76.34% for fracturing flowback fluid. Repeated usage and recycling have minimal effect on the photocatalyst TN_55_. The photocatalyst TN_55_ exhibits a high degree of stability and demonstrates excellent efficiency in the degradation of fracturing flowback fluid when it is recycled and reused.

**Figure 18:**
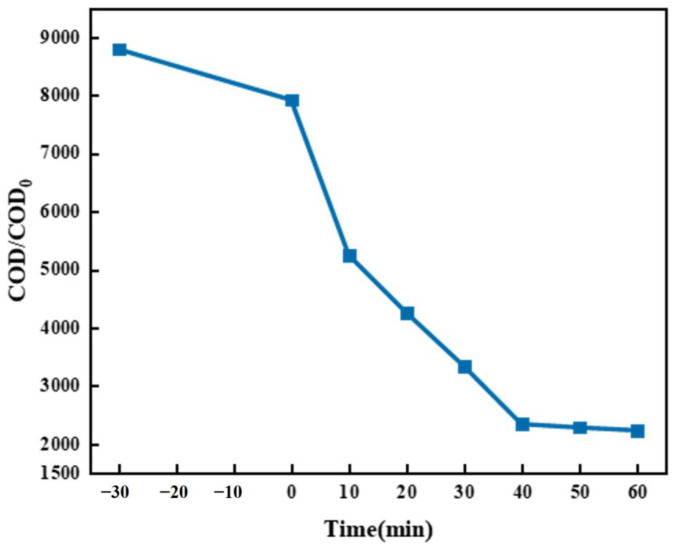
The adsorption capacity of TN_55_.

According to the figure’s results (Figure 18), during the dark adsorption stage (−30–0 min), the non-illumination adsorption process of the TiO_2_/Ni(OH)_2_ composite system is as follows: The initial COD value is 8798 (at −30 min), dropping to 7923.38 after 30 min (at 0 min). The absolute adsorption capacity reaches 874.62 COD units, with a removal rate of 9.94% and an average adsorption rate of 29.15 COD units/min. The lamellar structure of α-Ni(OH)_2_ captures pollutant molecules via Van der Waals forces, offering abundant adsorption sites. Hydroxyl groups (-OH) on the composite’s surface form hydrogen bonds with pollutants’ polar groups (e.g., -COOH, -NH_2_). Additionally, electrostatic adsorption may also occur.

**Figure 19:**
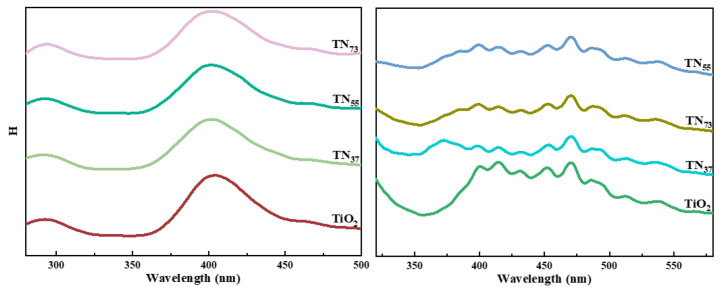
PL plot for different samples.

As illustrated in the figure (Figure 19), the luminescence intensity produced by the Ti/Ni composite when exposed to fluorescence at the same wavelength is lower than that of both TiO_2_ and α-Ni(OH)_2_. Additionally, the order of luminescence intensity from higher to lower is α-Ni(OH)_2_ > TiO_2_ > TiO_2_/α-Ni(OH)_2_. This suggests that the photogenerated electrons and holes of the TiO_2_/α-Ni(OH)_2_ composite photocatalysts have the lowest rate of recombination with photons. The compounding rate of the two substances is minimal, while the separation efficiency is significant, leading to a more potent oxidation performance. On the other hand, α-Ni(OH)_2_ displays the highest level of photoluminescence intensity. The combination of photogenerated electrons and holes in this material generates the largest number of photons. As a result, the photocatalytic oxidation performance of α-Ni(OH)_2_ is the least effective. The luminescence intensities of the TiO_2_/α-Ni(OH)_2_ samples, synthesized with varying mass ratios, were rated in the following order from highest to lowest: TN_37_, TN_73_, TN_55_. The fact that TN_55_ had the lowest photoluminescence intensity indicates that it performs the best in terms of degradation and that its COD/COD_0_ can reach 80.66%.

**Figure 20:**
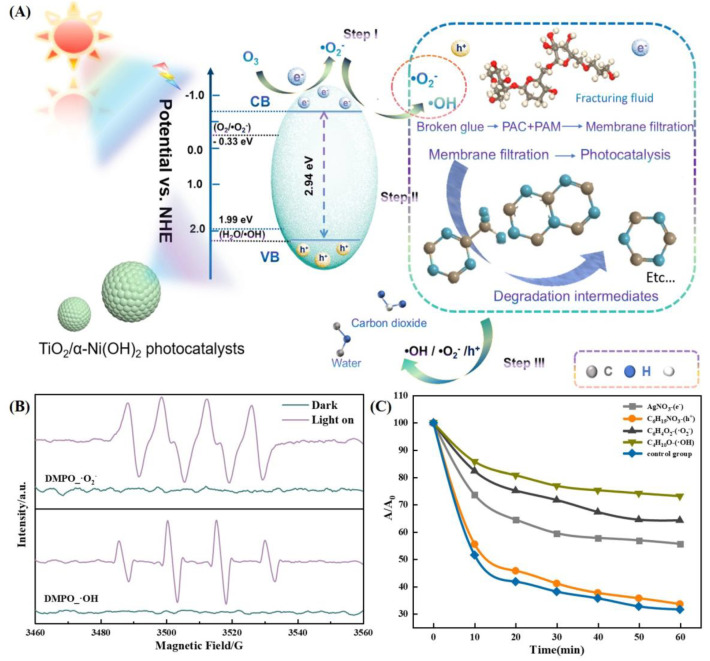
(**A**) Synergistic ozone-producing active species mechanism. (**B**) ESR test of TN_55_. (**C**) Photocatalytic activity with different trapping agents.

As illustrated in the figure (Figure 20A,C), the existence of -OH, -O_2_^−^, e^−^, and h^+^ species are involved in the photocatalytic degradation of fracturing flowback fluid by TiO_2_/α-Ni(OH)_2_. Nevertheless, the photogenerated hole (h^+^) species seems to have a negligible influence. The relative significance of the different categories of activities can be arranged in the following descending order: The hierarchy is as follows: The reaction pathway can be described as follows: The hydroxide ion (-OH) is converted to the superoxide ion (-O_2_^−^), which then releases an electron (e^−^) and a proton (h^+^). The proposed process for the photocatalytic degradation of fracturing flowback fluid using TiO_2_/α-Ni(OH)_2_ is as follows: When exposed to xenon lamp irradiation, the TiO_2_ particles on the surface of the TiO_2_/α-Ni(OH)_2_ photocatalyst get excited. The TiO_2_/α-Ni(OH)_2_ photocatalyst was initially stimulated, causing the migration of electrons (e) from the valence band (VB) to the conduction band (CB). Within the valence band (VB), electrons undergo a transition to the conduction band (CB), resulting in the creation of photogenerated holes (h^+^) within the initial valence band. Some of the electrons and holes will combine again, while the remaining electrons created by light (e^−^) will go to various locations on the surface of the TiO_2_/α-Ni(OH)_2_ photocatalyst. Under the influence of the electric field force, the electrons on the surface of the TiO_2_/α-Ni(OH)_2_ photocatalysts will move to different positions and contact with solvated O or adsorbed oxides on the surface. This interaction will result in the generation of superoxide radicals (-O_2_^−^). The superoxide radical (-O_2_^−^) and the hydroxyl radical (-OH) are produced through the reaction with dissolved oxygen or oxides that are attached to the surface of the photocatalyst. In addition, the presence of oxygen vacancies on the surface of α-Ni(OH)_2_ facilitates the formation of superoxide radicals. Afterwards, the interaction between photogenerated electrons and adsorbed dissolved oxygen or oxides on the photocatalyst’s surface leads to the production of superoxide radicals (-O_2_^−^) and hydroxyl radicals (-OH). To verify the theoretical deductions of thermodynamics, we performed an Electron Spin Resonance (ESR) test on TN_55_ to detect reactive oxygen species in Photocatalysis. The presence of ·OH and ·O_2_^−^ was observed when exposed to visible light (Figure 20B), indicating their generation under photocatalytic conditions; this discovery aligns with the expected generation of reactive oxygen species during the photocatalytic activity. These radicals have potent oxidizing properties, which aid in the breakdown of complex organic contaminants in the fracturing flowback fluid into simpler organic molecules or harmless substances.

## 3. Materials and Methods

### 3.1. Real Fracturing Flowback Fluid Composition Detection

The pollutant being studied in this inquiry is the fracturing flowback fluid that comes from the 11-well area of the Sulige gas field. The commonly employed indicators for water quality testing, as outlined in the Environmental quality standards for surface water (GB 3838-2002) [41], consist of pH, suspended substance solids (SS), chemical oxygen demand (COD), five-day biochemical oxygen demand (BOD_5_), and various salt ion concentrations. Evaluate the previously listed indicators in the study. (Note: COD/COD_0_ and SS/SS_0_ represent the corresponding removal rates of COD and SS.) COD_0_ represents the initial measurement of chemical oxygen demand (COD), while SS_0_ represents the initial measurement of suspended solids (SS)).

The SS of the fracturing flowback fluid was determined using a Clever Chem 380 Water Quality Analyzer, which is produced by DeChem-Tech in Hamburg, Germany. The COD of the fracture flowback fluid was measured using a versatile rapid dissolving device and a COD detector produced by Henan Suijing Environmental Protection Science and Technology Co. (Luoyang, China). The SQ-K80 fast BOD meter, produced by Shangqing Technology Co., Ltd. (Liuyang, China), was used to evaluate the BOD_5_ of the fracturing flowback fluid. An ion chromatograph from Aptar Ltd. (Crystal Lake, IL, USA) is used to analyze the inorganic salt ions present in fracturing flowback fluids. The ions to be measured are sodium (Na^+^), potassium (K^+^), calcium (Ca^+^), magnesium (Mg^2+^), chloride (Cl^−^), nitrate (NO_3_^−^), and sulphate (SO_4_^2−^). Before performing ion chromatographic analysis, it is necessary to filter the sample using a microporous organic filter membrane with a pore size of 0.2 μm. This procedure is essential for eliminating suspended pollutants and preventing the column from getting obstructed.

**Figure 21:**
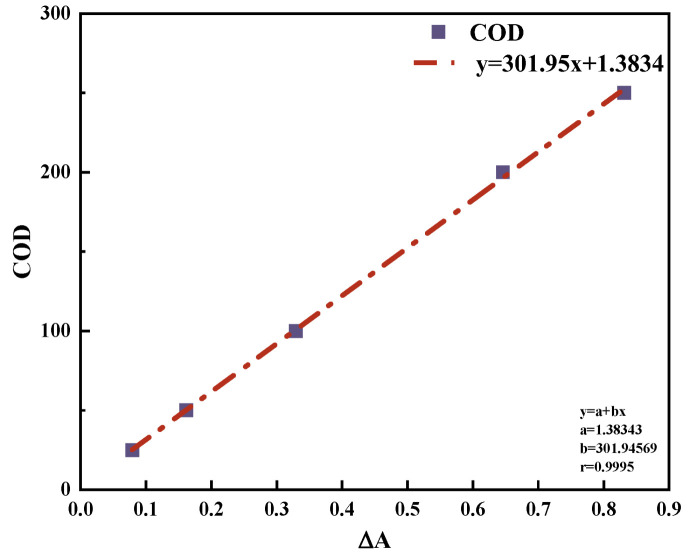
COD standard curve.

The COD calibration curve was constructed using potassium hydrogen phthalate solutions. A linear regression analysis yielded a correlation coefficient (r) of 0.9995, confirming the method’s accuracy (Figure 21).

The results are presented in Table 4. From the table, it can be seen that the pH value of the field-collected fracturing flowback fluid in this study was 6.8, slightly acidic. The fluid had an extremely high SS content of 896 mg/L, and its COD ranged from 8600 to 8800 mg/L, indicating a high organic matter concentration. Additionally, the fluid contained a high concentration of inorganic salt ions. The BOD_5_/COD ratio was approximately 0.015, less than 0.1, thus making it extremely difficult to biodegrade.

### 3.2. Synthesis Method of Photocatalyst

For this investigation, we chose to use hydrothermal synthesis to create pure anatase crystalline TiO_2_ nanoparticles, which act as photocatalysts [42,43,44,45]. Initially, 7 g of titanium sulphate (Ti(SO_4_)_2_) and 50 mL of deionized water were combined in a beaker. The beaker was then placed in a water bath set at a temperature of 60 °C, while the mixture was continuously stirred until the medication was thoroughly blended. Next, a certain quantity of NH_4_OH (Synthesized α-Ni(OH)_2_ is αα-Ni(OH)_2_/NO_3_^−^, without worrying about N interference) with a 20% concentration was employed to modify the pH of the titanium sulphate solution to 7. Throughout this time frame, a magnetic stirrer was used consistently for a duration of 2 h while keeping the temperature at 70 °C. After the solution has been stirred, it should be left to cool down to the temperature of the surrounding environment. For centrifugal washing, it is recommended to use deionized water and anhydrous ethanol in alternating cycles. Repeat this process 6 h. This will lead to the creation of a substance with a gel-like consistency that is white. Subsequently, a specific amount of H_2_O_2_ should be introduced directly into the gelatinous precipitate while simultaneously stirring and subjecting it to ultrasonication. The reaction will result in the formation of a translucent solution with an orange-yellow color, which will take approximately 2–3 h. The solution should be mixed with deionized water in a ratio of 1:5 and then transferred to a hydrothermal synthesis reactor with a PTFE coating on the inner surface. It is important to ensure that the liquid level does not exceed 70% of the reactor’s height. Subsequently, the reactor ought to be positioned within an electrically powered, temperature-controlled blast drying oven, with the temperature set precisely at 120 °C and maintained at this level for a duration of 10 h. Following the period of heat preservation, the reactor should be gradually cooled to the ambient temperature, resulting in the formation of a light blue translucent solution. Afterwards, the solution was freeze-dried using a lyophilizer, which yielded a white TiO_2_ sample powder after grinding.

Hydrothermal synthesis was chosen for the preparation of α-Ni(OH)_2_ [18].

For this investigation, we chose to use hydrothermal synthesis to create nano-TiO_2_/α-Ni(OH)_2_ photocatalysts. Initially, 0.7 g of pre-existing anatase TiO_2_ nanopowder was introduced into the solution. A 50 mL clean beaker was used to hold 3 g of α-Ni(OH)_2_ powder. Then, 40 mL of deionized water were added to the beaker. The mixture was then subjected to ultrasonication for a duration of 30 min until a uniform solution was achieved. Next, the solution was transferred to a hydrothermal synthesis reactor with a volume of 70 mL and subjected to a baking process in an oven at a temperature of 120 °C for a duration of 4 h. Following the baking process, the sample solution was extracted and then underwent centrifugation for a duration of 5 min at a speed of 8000 rpm. The method was iterated six cycles, employing anhydrous ethanol and deionized water as alternating solvents. The green colloidal sample was subsequently subjected to a thermal treatment in an electric oven at a temperature of 65 °C for a duration of 12 h, aiming to generate a solid green block sample. Ultimately, the sample was uniformly pulverized to produce a composite photocatalyst consisting of TiO_2_ and α-Ni(OH)_2_. The synthesis of TiO_2_/α-Ni(OH)_2_ composite photocatalysts with a mass ratio of 1:1 and 3:7 was also conducted using the same procedure. The obtained samples were labelled as TN_x_ (x = 73, 55, 37), with x being the synthesized mass ratio of TiO_2_ to α-Ni(OH)_2_.

### 3.3. Experimental Methods for Each Process

#### 3.3.1. Experimental Methods for Ozone Aeration Steps

**Figure 22:**
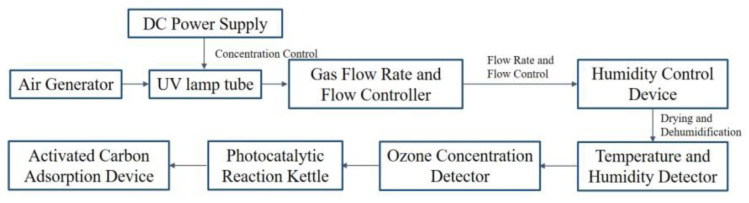
Schematic Diagram of Ozone Aeration Process.

Install the ozone aeration unit first. As seen in Figure 22, connect the air generator, UV lamp tube, DC power supply, gas flow rate and flow controller, dehumidifier, temperature and humidity detector, ozone concentration detector, and activated carbon adsorption device using hoses. Adjust the voltage of the UV lamp’s DC power source to control the ozone concentration and use the gas flow rate and flow controller to control the ozone flow rate and volume. To guarantee consistent aeration, the ozone aeration device uses an aeration disk.

With aeration time, concentration, and flow rate as single factors explore their impacts on flowback fluid degradation. Design concentration gradients of 20/40/60/80/100 ppm and flow rate gradients of 0.5/1/1.5/2 L/min. Measure 100 mL of fracturing flowback fluid at 25 °C. Set up an experimental group and an air control group, react for 60 min, and take two samples every 10 min for each group.

#### 3.3.2. Experimental Methods for Flocculating Settling Steps

PAM was selected as the coagulant aid to investigate the effects of different flocculants, pH, dosage, time, and agitation intensity. For flocculant optimization, 100 mL of homogenized fracturing flowback fluid was placed in five beakers, and 1 g/L of PAS, PFS, PFC, and PAC flocculants were added, respectively. The mixture was stirred at 150 r/min using a magnetic stirrer, followed by adding 0.1 g/L PAM. The rotation speed was adjusted to 150 r/min for 60 s of stirring. After stirring, the samples were left to settle for 60 min. During settling, floc size, sedimentation velocity, sedimentation time, and supernatant clarity were recorded. After settling, the supernatant was collected to measure COD and SS.

For the single-factor pH experiment, 20% H_2_SO₄ and NaOH were used to adjust the pH values of each group for reaction and subsequent measurements. For dosage analysis, PAM gradients were set at 0.05/0.1/0.15 g/L, and the selected flocculant gradients were 1/1.5/2 g/L. Optimization was conducted through nine groups of experiments with different ratios. For the single-factor sedimentation time experiment, fixed the remaining conditions and extended the reaction time to 90 min. Supernatant samples were taken every 10 min for SS and COD measurements. For the single-factor agitation intensity experiment, gradients of 50/100/150/200/250 r/min were set, and COD and SS were measured.

#### 3.3.3. Experimental Methods for Microporous Filter Membrane Steps

In this study, a hydrophilic mixed cellulose esters (MCE) membrane filter with a pore size of 0.1 μm was used for filtration, and a glass suction filtration apparatus was employed during the process. For experiments, 50 mL of homogenized raw fracturing flowback fluid was filtered through a 0.1 μm microporous membrane for compositional analysis.

#### 3.3.4. Experimental Methods for Photocatalytic Steps

For single-factor optimization, the experimental procedures were similar to those described in the previous section. When pH was the single factor, a gradient of 3/4/5…9 was set. For photocatalyst dosage optimization, dosage gradients of 0.2/0.4/0.6…2 g/L were designed. To investigate the synergistic degradation effect of ozone aeration on photocatalysis, the experimental steps were as follows: 100 mL of on-site fracturing flowback fluid was adjusted to pH 8 using 20% NaOH, and 0.12 g/L TN_55_ photocatalyst was added. An ozone aeration disc was then placed into the photocatalytic reaction kettle and connected to an aeration pipeline. The kettle was sealed with a quartz cover after confirming airtightness. A hose connected the kettle to an ozone generator, and another hose from the kettle’s gas outlet was linked to an activated carbon adsorption device to treat overflowing ozone. The voltage was adjusted to maintain an ozone concentration of 40 ppm at a flow rate of 1 L/min. Once abundant bubbles appeared on the liquid surface, the xenon lamp light source was activated, and positioned 10 cm above the liquid surface. Photocatalytic reactions were timed, and samples were taken every 10 min for testing.

#### 3.3.5. Experimental Methods for Substitutability Study

Experimental steps for single-factor optimization of electrochemical oxidation are as follows: Electrode plate type optimization: Select commonly used RuO_2_/Ti, Pt-Ir/Ti, and graphite plates as anodes for electrochemical oxidation. With an electrode spacing of 1 cm and a current of 0.5 A, 100 mL of flocculated, sedimented, and fine-filtered fracturing flowback fluid was electrochemically oxidized for 2 h. COD was measured after the reaction. Electrolysis time optimization: With an electrode spacing of 1 cm and a current of 0.5 A, fluid was treated for 6 h. Samples were taken every 1 h to monitor COD changes. Electrode spacing optimization: Set spacing gradients at 0.5/1/1.5/2 cm. Current intensity optimization: Set current gradients at 0.1/0.2/0.3…1.0 A and compare COD values.

The experimental steps for the single-factor optimization of Fenton oxidation are as follows: Optimization of Fenton oxidation time: Use the on-site fracturing flowback fluid that has been treated with the optimal flocculation and sedimentation parameters and then finely filtered for the experiment. Take 100 mL of the above-mentioned liquid and place it in a constant-temperature water bath to keep the temperature at a constant 25 °C during the reaction. Then, add 5 g/L of FeSO₄·7H_2_O and 50 mL/L of 30% H_2_O_2_ respectively, and stir the mixture at a speed of 150 r/min for 60 s. Start timing after the stirring is completed. Carry out oxidative degradation for 90 min. After allowing the liquid to stand for sedimentation for 60 min, take the supernatant for COD detection. Optimization of Fenton oxidation pH: First, add 5 g/L of FeSO₄·7H_2_O, and then add 50 mL/L of H_2_O_2_. Set the reaction time to 60 min. After the reaction is finished, let the mixture stand for 60 min, and then measure the COD of the supernatant. Optimization of Fenton oxidation reagent dosage: Similar to the experiment on the dosage of flocculation and sedimentation reagents, the dosages of two chemicals need to be discussed here. Set the dosage of FeSO_4_ at 4/5/6 g/L and the dosage of H_2_O_2_ at 40/50/60 mL/L. Conduct nine groups of experiments to determine the optimal values.

### 3.4. Laboratory Equipment and Chemicals

The laboratory equipment and chemicals required for this study are listed in the table below (Table 5 and Table 6).

## 4. Conclusions

In this study, TiO_2_/α-Ni(OH)_2_ photocatalytic composites were synthesized, and a flocculation and sedimentation-microfilm filtration-ozone synergistic photocatalytic process was established for the degradation of real fracturing flowback fluid.

(1) Coral-like anatase TiO_2_/α-Ni(OH)_2_ particles were synthesized using a hydrothermal method to enhance the photocatalytic performance and the ability to adsorb pollutants. The degradation effect was optimized when the mass ratio of Ti/Ni was 1:1.
(2) The 90 min photodegradation treatment system, which combines flocculation and sedimentation, membrane separation, and ozone synergism, can produce a degradation rate of 98.42% for the original fluid return. This degradation rate is 24.18% greater than that achieved by single-material catalytic treatment. This approach efficiently substitutes the procedures of evaporation, concentration, and electrocatalytic degradation in the initial treatment phase.
(3) The results of this study can provide valuable insights for the development and improvement of methods used to treat and optimize the discharge of shale gas fracturing.

## Figures and Tables

**Table 1:** Table of orthogonal experimental design and analysis for ozone aeration.

Number	Factors		
	A	B	C
	Time (min)	Concentration (ppm)	Current Velocity (L/min)
1	30	20	0.5
2	40	40	1
3	50	60	1.5
COD/COD_0_ range	3.25	6.32	3.6

**Table 2:** Table of orthogonal experimental design and analysis for Ti/Ni composite material photocatalysis.

Number	Factors		
	A	B	C
	Time (min)	pH	TN_55_ (g/L)
1	70	7	1
2	80	8	1.2
3	90	9	1.4
COD/COD_0_ range	2.77	1.93	3.7

**Table 3:** Composition table of fracturing flowback fluid.

	Processing Steps	Not	Flocculating Settling	Membrane Filtration	Complete
Composition					
pH		6.8	7	7	8
COD		8796	2551	878	135
SS		896	15	10	10
BOD_5_		67	-	-	17
Na^+^		4812	2332	2335	2312
K^+^		981	364	362	361
Ca^2+^		723	473	477	466
Mg^2+^		336	256	245	247
Cl^−^		7351	3768	3782	3714
SO_4_^2−^		326	251	235	264
NO_3_^−^		69	34	38	35

**Table 4:** Composition of fracturing flowback fluid.

Composition	Numerical Value (mg/L)	Composition	Numerical Value (mg/L)
pH	6.8	Ca^2+^	723
COD	8796	Mg^2+^	336
SS	896	Cl^−^	7351
BOD_5_	67	SO_4_^2−^	326
Na^+^	4812	NO_3_^−^	69
K^+^	981		

**Table 5:** List of laboratory equipment.

Name of Instrument	Models	Manufacturer
Electronic balances	YJ-DTF	Shanghai Yajin Electronic Technology Co., Ltd. (Shanghai, China)
Magnetic Heating Stirrers	HJ-6	Jinshui Yidu Instruments Co., Ltd. (Jinshui, China)
Electric Thermostatic Drying Oven	DHG-9030A	Shanghai Niyue Instrument Co., Ltd. (Shanghai, China)
Electric Thermostatic Water Bath	DZKW-4	Beijing Optical Century Instrument Co., Ltd. (Beijing, China)
Vacuum Drying Oven	DZF-6050	Shanghai Niyue Instrument Co., Ltd. (Shanghai, China)
Benchtop High-Speed Centrifuge	TG16G	Hunan Kaida Scientific Instrument Co., Ltd. (Changsha, China)
300 W Long-Arc Xenon Lamp	GXZ300	Shanghai Jiguang Special Lighting Electric Appliances Factory (Shanghai, China)
70 mL Hydrothermal Synthesis Autoclave	KH-70	Shanghai Zhize Biotechnology Development Co., Ltd. (Shanghai, China)
High-Pressure Quartz-Windowed Photocatalytic Reactor	PQ253	Beijing Perfectlight Technology Co., Ltd. (Beijing, China)
Multifunctional Rapid Digestion System	SJ-16X	Henan Suijing Environmental Technology Co., Ltd. (Luoyang, China)
Chemical Oxygen Demand (COD) Spectrophotometric Analysis System	GNST-900	Henan Suijing Environmental Technology Co., Ltd. (Luoyang, China)
Multi-Purpose X-ray Diffractometer (XRD) System	X’pertPRO	Netherlands PANalytical (Almelo, The Netherlands)
X-ray Photoelectron Spectrometer (XPS)	Thermo-Scientific TM ESCALAB250Xi	Thermo Fisher Scientific (Waltham, MA, USA)
Ultraviolet-Visible Diffuse Reflectance Spectrometer (UV-Vis DRS)	UV-2600	Japan SHIMADZU (Kyoto, Japan)
High-Resolution Field Emission Scanning Electron Microscope (HR FE-SEM)	Gemini Sigma360	German Carl Zeiss AG (Oberkochen, Germany)
Scanning Electron Microscope (SEM)	SU8010	Japan Hitachi (Chiyoda, Japan)
pH Meter	PHS-25	Shanghai Yuefeng Instruments and Meters Co., Ltd. (Shanghai, China)
Ion Chromatograph (IC)	881	Swiss Metrohm (Herisau, Switzerland)
Spectrophotometric Water Quality Analyzer	CleverChem 380	German DeChem-Tech. GmbH (Hamburg, Germany)
BOD Rapid Analyzer	SQ-K80	Shangqing Technology Co., Ltd. (Shanghai, China)
Thermostatic Magnetic Stirring Water Bath	DF-101S	Gongyi Yuhua Instruments Co., Ltd. (Gongyi, China)
Hydrophilic Mixed Cellulose Esters (MCE) Membrane Filter	/	Delvstlab (Jiaxing, China)
250 mL Sintered Glass Filter Assembly	/	Gaode Glassware Co., Ltd. (Wenzhou, China)
Programmable DC Power Supply	MS-6050	Dongguan Maihao Electronic Technology Co., Ltd. (Dongguan, China)
High-Precision UV Photometric Ozone Analyzer	2B 106 L	Beijing Tonglin Technology Co., Ltd. (Beijing, China)
Laboratory-Grade Zero Air Generator	OML-3000A	Sichuan Oumeili Technology Co., Ltd. (Chengdu, China)
UV-C Disinfection Lamp	225-VUV (185 nm)	Guangzhou Langpu Optoelectronic Technology Co., Ltd. (Guangzhou, China)
High-Precision Thermal Mass Flow Meter	CX-GMFM	Shanghai Jishen Instruments and Meters Co., Ltd. (Shanghai, China)

**Table 6:** List of laboratory chemicals.

Reagent Name	Grade	Manufacturer
Ni(NO_3_)_2_·6H_2_O	AR	Shanghai Aladdin Biochemical Technology Co., Ltd. (Shanghai, China)
CH₄N_2_O	AR	Shanghai Aladdin Biochemical Technology Co., Ltd. (Shanghai, China)
CH_3_CH_2_OH	AR	Shanghai Aladdin Biochemical Technology Co., Ltd. (Shanghai, China)
Ti(SO_4_)_2_	AR	Chengdu Kelong Chemical Reagents Factory (Chengdu, China)
H_2_O_2_	AR	Chengdu Kelong Chemical Reagents Factory (Chengdu, China)
* GW-3	T.P.	Chengdu Kelong Chemical Reagents Factory (Chengdu, China)
XLW-32	T.P.	Chengdu Kelong Chemical Reagents Factory (Chengdu, China)
XLW-30G	T.P.	Chengdu Kelong Chemical Reagents Factory (Chengdu, China)
Magnacide575	T.P.	Chengdu Kelong Chemical Reagents Factory (Chengdu, China)
Claytreat-3C	T.P.	Chengdu Kelong Chemical Reagents Factory (Chengdu, China)
Claymaster-5C	T.P.	Chengdu Kelong Chemical Reagents Factory (Chengdu, China)
Inflo-251G	T.P.	Chengdu Kelong Chemical Reagents Factory (Chengdu, China)
BF-7L	T.P.	Chengdu Kelong Chemical Reagents Factory (Chengdu, China)
BC-31	T.P.	Chengdu Kelong Chemical Reagents Factory (Chengdu, China)
* GBW-5	T.P.	Chengdu Kelong Chemical Reagents Factory (Chengdu, China)
PAC	T.P.	Chengdu Kelong Chemical Reagents Factory (Chengdu, China)
PFS	T.P.	Chengdu Kelong Chemical Reagents Factory (Chengdu, China)
PAS	T.P.	Chengdu Kelong Chemical Reagents Factory (Chengdu, China)
PFC	T.P.	Chengdu Kelong Chemical Reagents Factory (Chengdu, China)
PAM	T.P.	Chengdu Kelong Chemical Reagents Factory (Chengdu, China)
H_2_SO_4_	AR	Chengdu Kelong Chemical Reagents Factory (Chengdu, China)
HgSO_4_	AR	Chengdu Kelong Chemical Reagents Factory (Chengdu, China)
K_2_Cr_2_O_7_	AR	Chengdu Kelong Chemical Reagents Factory (Chengdu, China)
Ag_2_SO_4_	AR	Chengdu Kelong Chemical Reagents Factory (Chengdu, China)
Raw Fracturing Flowback Fluid	/	Surig Gas Field(Ordos, China)
Fe_S_O_4_·7H_2_O	AR	Shanghai Aladdin Biochemical Technology Co., Ltd. (Shanghai, China)
AgNO_3_	AR	Shanghai Aladdin Biochemical Technology Co., Ltd. (Shanghai, China)
C_6_H_15_NO_3_	AR	Shanghai Aladdin Biochemical Technology Co., Ltd. (Shanghai, China)
C_6_H_4_O_2_	AR	Shanghai Aladdin Biochemical Technology Co., Ltd. (Shanghai, China)
C_4_H_10_O	AR	Shanghai Aladdin Biochemical Technology Co., Ltd. (Shanghai, China)
C_8_H_5_O_4_K	SP	Shanghai Aladdin Biochemical Technology Co., Ltd. (Shanghai, China)

## Data Availability

The raw data supporting the conclusion of this article will be made available by the authors, without undue reservation.

## Supplementary Materials

The following supporting information can be downloaded at: https://www.mdpi.com/article/10.3390/molecules30071568/s1, The raw SEM images.

## Abbreviations

The following abbreviations are used in this manuscript:

COD	Chemical Oxygen Demand
BOD	Biochemical Oxygen Demand
SS	Suspended Solids
PTFE	Polytetrafluoroethylen
XRD	X-ray Diffraction
SEM	Scanning Electron Microscopy
XPS	X-ray Photoelectron Spectroscopy
BJH	Barrett-Joyner-Halenda method
ESR	Electron Spin Resonance
